# Colorectal cancer in ulcerative colitis after liver transplantation for primary sclerosing cholangitis: a systematic review and pooled analysis of oncological outcomes

**DOI:** 10.1007/s12672-024-01304-6

**Published:** 2024-10-08

**Authors:** Roberta Angelico, Leandro Siragusa, Francesca Blasi, Vittoria Bellato, Michela Mineccia, Elisabetta Lolli, Giovanni Monteleone, Giuseppe S. Sica

**Affiliations:** 1 HPB and Transplant Unit, Department of Surgical Sciences, University of Rome “Tor Vergata”, Rome, Italy; 2 Division of Colon and Rectal Surgery, IRCCS Humanitas Research Hospital, Rozzano, Milan Italy; 3 Minimally Invasive and Digestive Surgery Unit, Department of Surgical Sciences, University of Rome “Tor Vergata”, Rome, Italy; 4 Azienda Ospedaliera Ordine Mauriziano di Torino, Turin, Italy; 5 Department of Systems Medicine, University of Rome “Tor Vergata”, Rome, Italy

**Keywords:** Colorectal cancer, Liver transplantation, Ulcerative colitis, Primary sclerosing cholangitis

## Abstract

**Introduction:**

Patients with ulcerative colitis (UC) receiving liver transplantation (LT) due to primary sclerosing cholangitis (PSC) have higher risk of developing colorectal cancers (CRC). Aim of this systematic review was to define the patients’ features, immunosuppressive management, and oncological outcomes of LT recipients with UC-PSC developing CRC.

**Methods:**

Searches were conducted in PubMed (MEDLINE), Cochrane Library, Web of Science for all English articles published until September 2023. Inclusion criteria were original articles including patients specifying outcomes of interest. Primary endpoints comprised incidence of CRC, disease free survival (DFS), overall survival (OS) and cancer recurrence. Secondary endpoints were patient’s and tumor characteristics, graft function, immunosuppressive management and PSC recurrence. PROSPERO CRD42022369190.

**Results:**

Fifteen studies included, 88 patients were identified. Patients (mean age: 50 years) had a long history of UC (20 years), mainly with active colitis (79%), and developed tumor within the first 3 years from LT, while receiving a double or triple immunosuppressive therapy. Cumulative incidence of tumor was 5.5%. At one, two and three years, DFS was 92%, 82% and 75%, while OS was 87%, 81% and 79% respectively. Disease progression rate was 15%. After CRC surgery, 94% of patients maintained a good graft functionality, with no reported cases of PSC recurrence.

**Conclusions:**

After LT, patients with PSC and UC have an increased risk of CRC, especially in presence of long history of UC and active colitis. Surgical resection guarantees satisfactory mid-term oncological outcomes, but samples are limited, and long-term data are lacking. National and international registry are auspicial to evaluate long-term oncological outcomes and to optimize clinical management.

## Introduction

Ulcerative colitis (UC) is an inflammatory bowel disease (IBD) characterized by inflammation of the rectum and colon, due to abnormal immune response. Primary sclerosing cholangitis (PSC) is a UC extraintestinal manifestation, characterized by multi-focal bile duct strictures and progressive cholestatic disease leading to liver cirrhosis [1–3]. In case of progression to end-stage liver disease, liver transplantation (LT) is the sole intervention with curative intention [4–6]. As matter of fact, PSC is the primary indication for LT in approximately 5% of all adult recipients and is associated with IBD in up to 70% of patients [7–9].

UC increases the risk of developing colorectal cancer (CRC) depending on the severity, extension, and duration of the colitis [10]. When UC is combined with PSC, the risk of tumor rises to tenfold [3, 11, 12]. Moreover, the risk of developing UC-associated CRC is enhanced after LT for PSC, possibly due to immunosuppression and a more aggressive course of the colitis. Furthermore, the post-transplant immunosuppressive (IS) therapy is a known risk factor for the development of de novo neoplasia, which represent a major cause of morbidity and mortality in LT recipients [10, 13].

Clinical management and oncological outcome of UC patients developing CRC after LT for PSC has been evaluated only by few retrospective studies. Recently, we outlined an algorithm to optimize the surgical approach for the treatment of CRC in UC patients after LT, but the optimal IS management, has not yet been clarified [14]. In PSC LT recipients, IS regimen usually follows the clinical guidelines designed for autoimmune liver diseases, but there's a lack of specific recommendations for optimizing IS for CRC prevention or management [15–18]. In fact, despite CRC is a leading cause of death in these patients, resourceful data are scarce, as recently reported by the European Society of Organ Transplantation Consensus [19].

The aims of this systematic review were to define the oncological outcome and immunosuppressive regimen of LT recipients with UC-PSC developing CRC.

## Materials and methods

A systematic research was conducted to identify relevant studies focused on the presence of CRC or dysplasia in LT recipients with UC and PSC. This systematic review complied with the PRISMA guidelines [20] and was registered in PROSPERO CRD42022369190.

### Search strategy

All articles published in English up until until September 2023 were searched. A systematic search within the PubMed (MEDLINE), Cochrane Library, Web of Science electronic databases was carried out to identify relevant English-language articles. The following term combination was used: “colorectal cancer”, “liver transplantation”, “ulcerative colitis”, and “primary sclerosing cholangitis”. Records were screened for relevance based on their title and abstract, then the full texts of the selected articles were retrieved and analysed. Furthermore, the references list of each eligible article was screened to identify possible additional relevant studies.

### Study selection

Eligible studies fulfilled the following inclusion criteria: (1) articles including adult LT recipients with UC-PSC affected by CRC or dysplasia; (2) original articles, clinical trials, case series and case reports; (3) articles specifying at least one outcome of interest. Exclusion criteria were as follow: (1) article that did not differentiate UC from Crohn’s disease; (2) unable to extract patients’ data from articles; (3) same patients series included in different studies.

### Data extraction and synthesis

Two authors (F.B. and L.S.) independently screened each record from full text articles for eligibility and extracted: study characteristics and design, number of patients and all information related to demographics and outcomes measures. Disagreement was resolved by discussion and consensus; if no agreement was reached, a senior author was consulted (G.S.S.).

### Outcome measures

Primary endpoints included incidence of CRC and oncological outcome in terms of disease-free survival (DFS), overall survival (OS) and disease progression. Secondary endpoints were patient’s and tumor characteristics, graft function, IS management and PSC recurrence.

The baseline characteristics of the studies comprised patients’ demographics (gender, age at UC and CRC diagnosis, colonoscopy after LT), transplant data, post-transplant IS regimen, and CRC features (type, TNM stage, site) [21].

### Study quality assessment

Methodological index for non-randomized studies (MINORS) was employed to measure the quality of non-randomized studies included in systematic reviews [22]. Non-comparative articles were assessed for 8 parameters, comparative one for 12 parameters, each awarding up to two points, with a maximum total score of 16 points for non-comparative and 24 for comparative.

### Statistical analysis

Categorical data were reported as absolute numbers with percentage, continuous data are reported as median with ranges. Data were pooled and descriptive statistics produced from the dataset. The number of patients to which each variable refers was defined as (#number). A pooled analysis was performed where categorical and continuous data were reported as mean, range and percentages. Individual patient data from the individual studies were used to construct the time-to-event Kaplan–Meier curves. There was no comparative statistical analysis.

## Results

### Systematic search

**Fig. 1:**
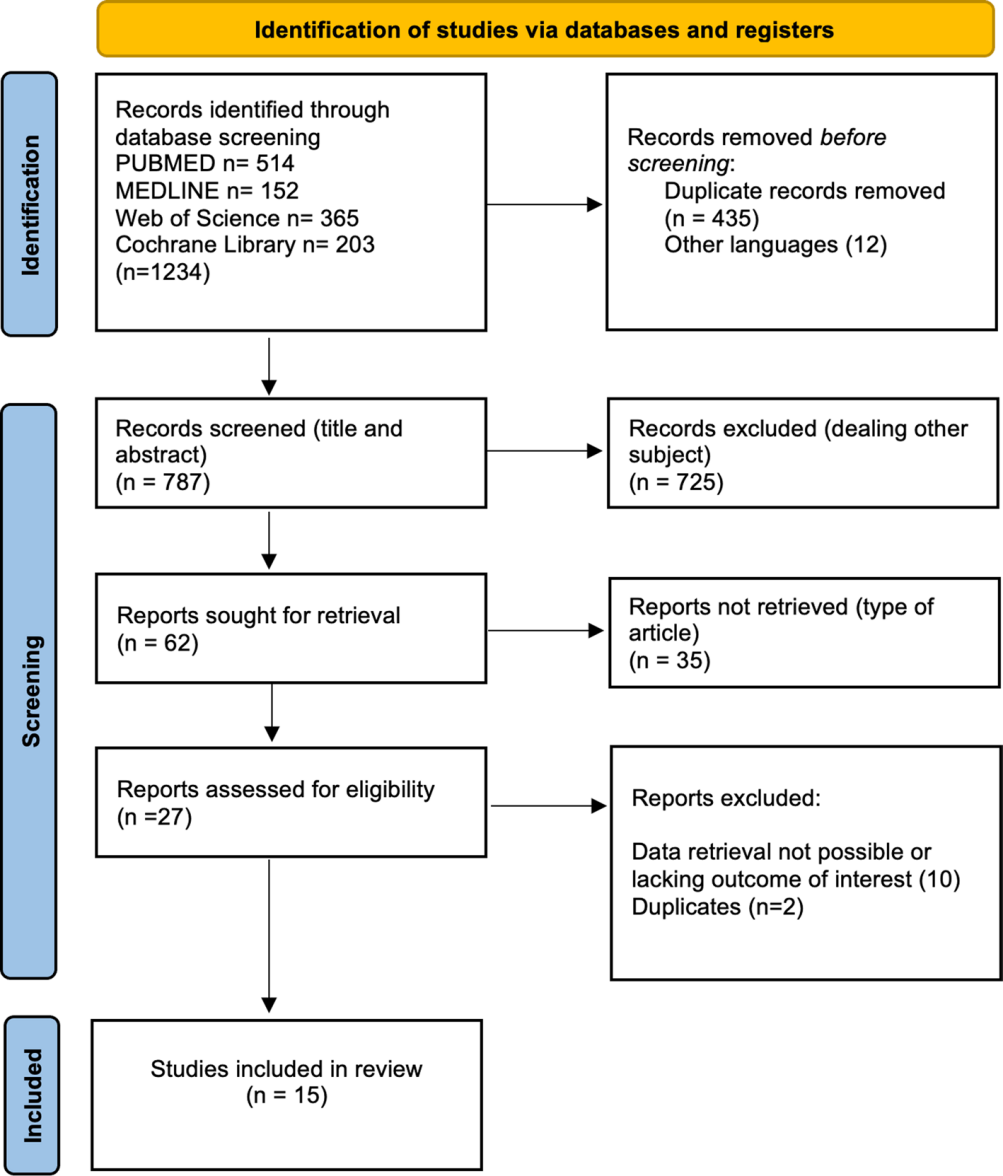
Systematic search process

Through the database systematic search, 1234 articles were identified as follows: 514 from PUBMED, 152 from MEDLINE, 365 from Web of Science and 203 from Cochrane Library. Out of the identified articles, 447 were eliminated (435 duplicates, 12 non-English language) as detailed in Fig. 1. After screening, 725 articles dealing with other subjects were also excluded. Of the remaining 27 eligible articles who underwent full-text reading, 12 articles were excluded owing to inability to retrieve patients’ data, lacking primary outcomes or inclusion of data from patients present in more than one study. Finally, 15 studies met the inclusion criteria and were selected for the systematic review [15, 16, 23–35].

### Study characteristics and quality assessment

Articles were published between 1990 and 2022, including eleven retrospective studies, three case report and one case series with a total of 88 patients. Average MINORS score was 8.8/16 for non-comparative studies and 13.6/24 for comparative studies. Characteristics of the studies included in the review are summarised in Table 1.

**Table 1:** Characteristics of the studies included in the review

Author	Year	City (Country)	Study design	Number of patients	MINORS score
Higashi et al. [15]	1990	Pittsburgh (USA)	Case series	2	9/16
Bleday et al. [23]	1993	Boston (USA)	Retrospective study	3	8/16
Knechtle et al. [16]	1995	Madison (USA)	Retrospective study	2	8/16
Narumi et al. [24]	1995	California (USA)	Retrospective study	2	8/16
Fabia et al. [25]	1998	Dallas (USA)	Retrospective study	5	12/24
Loftus et al. [26]	1998	Rochester (USA)	Retrospective study	9	10/16
MacLean et al. [27]	2002	Toronto (CA)	Retrospective study	2	9/16
Van de Vrie et al. [28]	2003	Rotterdam (NL)	Retrospective study	4	14/24
Vera et al. [29]	2003	Birmingham (UK)	Retrospective study	8	9/16
Bosso et al. [30]	2009	Torino (IT)	Retrospective study	3	9/16
Fukuhara et al. [31]	2009	Kyushu (JP)	CR	1	–
Horvath et al. [32]	2013	Tubingen (DEU)	CR	1	–
Obusez et al. [33]	2013	Cleveland (USA)	Retrospective study	18	15/24
Rompianesi et al. [34]	2018	London (UK)	Retrospective study	27	9/16
Miyagi et al. [35]	2020	Uehara (JP)	CR	1	–

MINORS: Methodological index for non-randomized studies

### Patient characteristics

Eighty-eight UC patients who developed CRC after LT due to PSC were identified. Ten studies (#29) provided information regarding patients’ gender (65% male). At LT, patients (#27) had a mean age of 47 years (range 21–58), while at CRC diagnosis patients (#29) the mean age was 50 years old (range 31–60). In ten studies (#34), 39% of patient were mentioned to have undergone a complete screening coloscopy after LT. Twelve studies (#41) reported that the mean time from LT to CRC diagnosis was 41 months (range 8–120). Prior CRC diagnosis, the mean duration of UC disease was 21 years (range 9–40) as reported by nine studies (#24). Seven studies (#28) reported the UC clinical status after LT including 47% patients with pancolitis, 32% with active colitis and 21% with quiescent colitis.

The post-transplant IS drugs were mentioned by eight studies (#19) and consisted in steroids (84%), Azathioprine (AZA) (68%), Cyclosporine (Cya) (63%), Tacrolimus (Tac) (16%), Mychophenolate Mofetil (MMF) (10%) and Rapamycin (5%). The IS regimen was based on three IS drugs in 16 (84.2%) patients, while on two IS drugs in 3 (15.8%) patients. Patients’ baseline characteristics are detailed in Table 2**.**

**Table 2:** Baseline characteristics

Author	n°	Sex n (%)	Mean age at LT (years)	Screening colonoscopy after LT n (%)	Mean time to CRC diagnosis post LT (months)	Mean age at CRC (years)	Mean time UC before CRC (years)	IS type post LT n (%)	Status of UC after LT n (%)
Higashi et al. [15]	2	1F (50%) 1 M (50%	46	2 (100%)	17 m	47.5	19	Cya + Aza	Quiescent colitis 2 (100%)
Bleday et al. [23]	3	1 F (33%) 2 M (67%)	43	3 (100%)	11 m	44	19.3	Cya + Aza + steroids	na
Knechtle et al. [16]	2	na	na	na	36 m	na	na	na	Active colitis 2 (67%) Quiescent 1 (33%)
Narumi et al. [24]	2	1F (50%) 1 M (50%)	58	na	21 m	60	40.5	Cya + Aza + steriods	na
Fabia et al. [25]	5	1F (20%) 4 M (80%)	44	5 (100%)	24 m	46	na	Cya + Aza + steroids	na
Loftus et al. [26]	9	na	na	9 (100%)	48 m	na	na	na	Pancolitis 9 (100%)
MacLean et al. [27]	2	1F (50%) 1 M (50%)	na	2 (100%)	na	53	23.5	na	na
Van de Vrie et al. [28]	4	2F (50%) 2 M (50%)	48	na	47 m	52	13.5	CNI + Aza + steroids	Pancolitis 4 (100%)
Vera et al. [29]	8	F 3 (38%) M 5 (62%)	53	8 (100%)	44 m	56.5	22.5	na	Active colitis 7 (87%) Quiescent colitis 1 (13%)
Bosso et al. [30]	3	na	na	3 (100%)	79 m	na	na	na	na
Fukuhara et al. [31]	1	1 M (100%)	49	1 (100%)	8 m	49	9	Tac + MMF + steroids	Quiescent colitis
Horvath et al. [32]	1	1 M (100%)	21	1 (100%)	120 m	31	na	Tac + Sir	Quiescent colitis
Obusez et al. [33]	18	na	na	na	na	na	na	na	na
Rompianesi et al. [34]	27	na	na	na	na	na	na	na	na
Miyagi et al. [35]	1	1 M (100%)	32	1 (100%)	0	32	20	Tac + MMF + steroids	na
Total	88	F 10/29 (34.5%) M 19/29 (65.5%)	Mean 47 years (mean range 21–58) 27pt	34/88 39%	Mean 40 months (mean range 8–120) 41pt	Mean 50 months (mean range 31–60) 29pt	Mean 21 years (mean range 9–40.5) 23pt	Steroids 16/19 (84.2%) Aza 13/19 (68.4%) Cya 12/19 (63.1%) MMF 2/19 (10.5%) Tac 3/19 (15.8%) Sirolimus 1/19 (5.2%)	Pancolitis 13/28 (46.5%) Active colitis 9/28 (32.1%) Quiescent colitis 6/28 (21.4%)

LT: liver transplant; CRC: colorectal cancer; UC: ulcerative colitis; IS: immunosuppressive therapy; F: female; M: male; m: months; Cya: Cyclosporine; Aza: Azathioprine; CNI: Calcineurin inhibitors; Tac; MMF: Mychophenolate Mofetile; pt: patients

### Tumor features and oncological outcomes

The incidence of CRC in LT recipients with UC-PSC was 5.5% as reported by eleven studies (#81). All studies (#88) described the histologic features of the tumor, including 67% CRC, 6% high-grade dysplasia (HGD), 7% low-grade dysplasia (LGD), 2% dysplasia-associated lesion or mass (DALM) and 18% unspecified dysplasia. Cancer localisation (#28) was observed in the right colon (25%), rectum (21%), left colon (14%), transverse colon (11%) or with synchronous neoplasia on different location (11%); in 18% of cases the localisation was unspecified. TNM staging was recorded by eleven studies (#44): stage 0 in 66% of patients, stage I in 14%, stage II in 4%, stage III in 7% and stage IV in 11% cases.

**Fig. 2:**
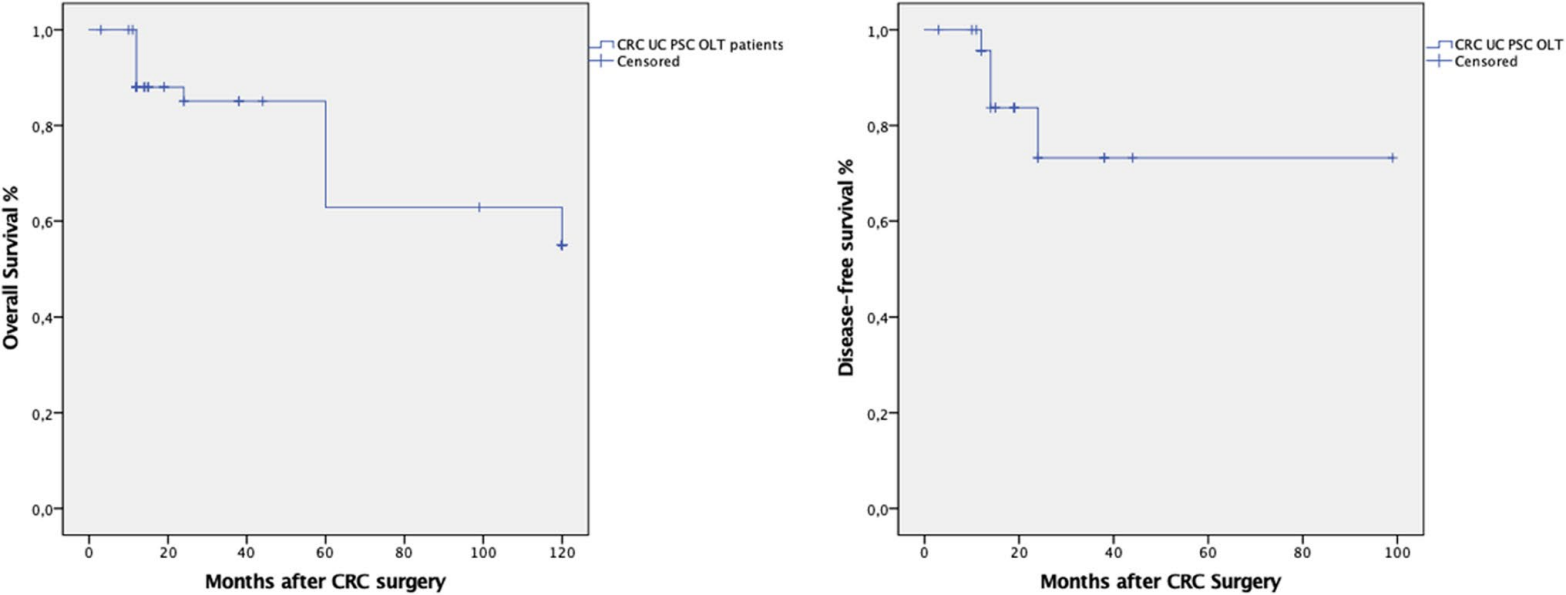
Overall survival and disease-free survival of liver transplant recipients with UC-PSC developing colorectal cancer

Nine studies (#26) provided information on CRC recurrence rate after treatment, which was observed in 15% of cases (7.5% liver and 7.5% unspecified). From the same studies, the DFS rates were 92% at 1-year (#26), 82% at 2-year (#11) and 75% at 3-year (#6). The mean time to disease progression was 25 months (range 12–38) (Fig. 2).

Nine studies (#53) reported on patient survivals: 1-year OS rate was 87% (#53), 2-year OS was 81% (#32) and 3-year OS was 79% (#28). The mean time from CRC to dead was 24 months (range 12–96), and mortality was cancer-related in 30% cases, while graft-related in 5% of cases (Fig. 2).

Graft functionality was recorded by seven studies (#18). At the last follow-up, 94% of patients maintained a good graft functionality, and only one (6%) patient developed chronic rejection. No case of PSC recurrence has been reported.

**Fig. 3:**
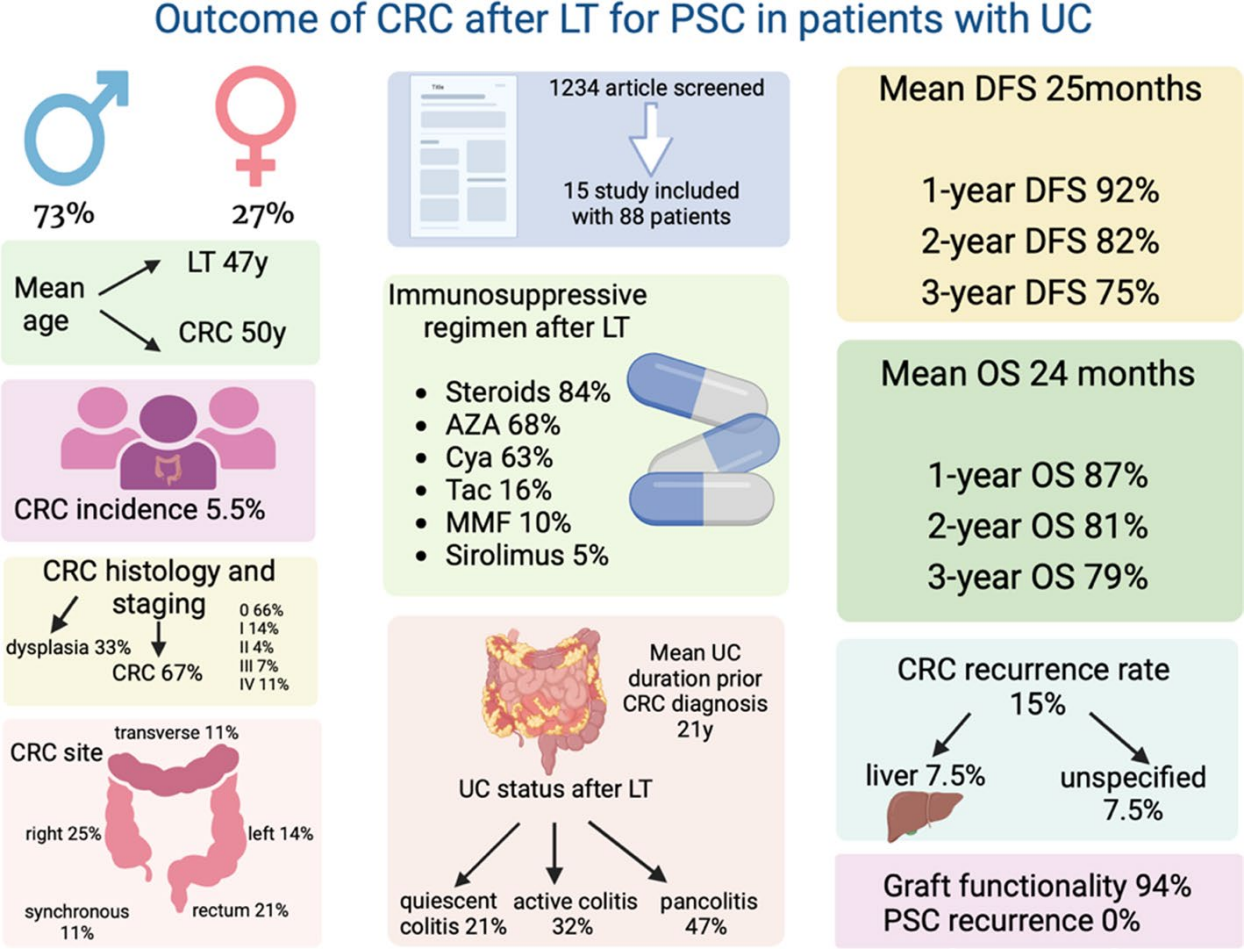
Outcomes colorectal cancer in liver transplant recipients for PSC and UC

Primary and secondary outcomes of UC patients developing CRC after LT for PSC are summarised in Table 3 and Fig. 3.

**Table 3:** Oncological and graft outcomes

Author	N° pts	Hystologic characteristic n (%)	CRC incidence n (%)	CRC site n (%)	TNM staging n (%)	CRC disease progression n (%)	Mean DFS (months)	Mean/Median OS (months)	Mortality n (%)	Length of follow-up (months)	Functionality of graft n (%)	PSC recurrence n (%)
Higashi et al. [15]	2	2 CRC (100%)	2/36 (6%)	Rectum 1 (50%) Severe dysplasia of the entire colon 1 (50%)	T3N +M0 1 (50%) T1N0M0 1 (50%9	0 (0%)	19 m	19 m	0 (0%)	19 m	Yes	0 (0%)
Bleday et al. [23]	3	2 CRC (67%) 1 HGD (33%)	3/32 (9%)	Hepatic flexure 1 (33%) Rectosigmoid junction 1 (33%) Rectum 1 (33%)	TxN0M0 1 (33.3%) TxN0M0 1 (33.3%) TisN0M0 1 (33.3%)	0 (0%)	38 m	38 m	0 (0%)	36 m	Yes	0 (0%)
Knechtle et al. [16]	2	2 CRC (100%)	2/29 (7%)	na	na	0 (0%)	12 m	12 m	0 (0%)	12 m	Yes	0 (0%)
Narumi et al. [24]	2	1 CRC (50%) 1 HGD (50%)	2/37 (5%)	Rectum 1 (50%) Unspecified colon 1 (50%)	TxN0M0 1 (50%) TisN0M0 1 (50%)	na	na	na	Dead 1 (50%)	na	na	na
Fabia et al. [25]	5	5 CRC (100%)	5/63 (8%)	Rectum 1 (20%) Unspecified colon 4 (80%)	na	unspecified site 2 (40%) no 3 (60%)	1 12 m 1 24 m 3 > 24 m	2 > 12 m 3 > 24 m	Cancer related 2 (40% =	24 m	na	na
Loftus et al. [26]	9	1 CRC (11%) 1 HGD (11%9 5 LGD (56%) 2 DALM (22%)	9/57 (15.8%)	na	TisN0M0 8 (89%) Uspecified 1 (11%)	0 (0%)	32 m	32 m	0 (0%)	32 m	na	na
MacLean et al. [27]	2	2 CRC (100%)	2/40 (5%)	Rectum 1 (50%) Right colon 1 (50%)	na	na	na	na	na	na	na	na
Van de Vrie et al. [28]	4	2 CRC (50%) 1 HGD (25%) 1 LGD (25%)	na	Right colon 1 (25%) Left colon 1 (25%) Synchronous right and left 2 (50%)	TxNxM1 1 (25%) TxN0M0 1 (25%) TisN0M0 2 (50%)	na	na	na	Cancer related 1 (25%)	na	na	na
Vera et al. [29]	8	8 CRC (100%)	8/83 (10%)	Right colon 3 (38%) Transverse colon 3 (38%) Left colon 2 (24%)	Stage 1 3 (38%) Stage 2 1 (12%) Stage 3 1 (12%) Stage 4 3 (38%)	na	na	na	Cancer related 3 (38%) Graft related 1 (12%)	na	Chronic rejection 1 (12%) yes 7 (88%)	0 (0%)
Bosso et al. [30]	3	2 CRC (67%) 1 HGD (33%)	3/16 (19%)	na	T3N0M0 (33.3%) T2N0M0 (33.3%) T1s N0M0 (33.3%)	Liver 2 (67%)	2 < 12 m 1 > 12 m	14 m	0	14 m	na	na
Fukuhara et al. [31]	1	1 CRC (100%)	na	Left colon 1 (100%)	TxN0M0 1 (100%)	na	na	na	0	na	yes	0 (0%)
Horvath et al. [32]	1	1 CRC (100%)	na	na	TxNxM1c1 (100%)	0 (0%)	12 m	12 m	0	12 m	yes	0 (0%)
Obusez et al. [33]	18	2 CRC (11%) 16 unspecified dysplasia (89%)	18/49 (37%)	na	TisN0M0 16 (89%) Unspecified 2 (11%)	na	na	na	na	na	na	na
Rompianesi et al. [34]	27	27 CRC 0 (100%)	27/677 (4%)	na	na	na	na	*96 m*	Dead 13 (48%)	na	na	na
Miyagi et al. [35]	1	1 CRC 0 (100%)	na	Right colon1 (100%)	T1N0M0 1 (100%)	0 (0%)	12 m	12 m	0	12 m	yes	0 (0%)
Total	88	CRC 59/88 67% HGD 5/88 5.7% LGD 6/88 6.8% DALM 2/88 2.3% Unspecified dysplasia 16/88 18.2%	81/1487 (5.5%)	Right colon 7/28 (25%) Transverse colon 3/28 (10.7%) Left colon 4/28 (14.3%) Rectum 6/28 (21.4%) Syncronous 3/28 (10.7%) Unspecified colon 5/28 (17.9%)	Stage 0 29/44 (65.9%) Stage 1 6/44 (13.7%) Stage 2 2/44 (4.5%) Stage 3 2/44 (4.5%) Stage 4 5/44 (11.4%)	No recurrence 22/26 (84.6%) Liver 2/26 (7.7%) Unspecified site 2/27 (7.7%)	Mean DFS 25.1 m 26pt 1y DFS 24/26 92.3% 2y DFS 9/11 81.8% 3y DFS 4/6 75%	Mean OS 24.1 m 1y OS 46/53 86.8% 2y OS 26/32 81.3% 3y OS 22/28 78.6%	Dead 21/68 (30.8%) Cancer-related 6/21 (28.6%) Graft-related 1/21 (4.7%) Unspecified 14/21 (66.6%)	Mean fu 25.5 m 26pt	Chronic rejection 1/18 (5.5%) Yes 17/18 (94.5%)	PSC recurrence 0/18 (0%)

PSC: primary sclerosing cholangitis; CRC: colorectal cancer; DFS: disease-free survival; OS: overall survival; HGD: high-grade dysplasia; LGD: low-grade dysplasia; DALM: dysplasia-associated lesion or mass; fu: follow-up; m: month, y: year, pt: patients

## Discussion

The current systematic review is the first reporting clinical characteristics of UC patients developing CRC after LT for PSC and describing clinical management and oncological outcomes. The reported incidence of CRC was 5.5%, with tumors presenting early after transplantation, mainly in adult patients with a long history of active UC. Data showed a good patients’ survival and tumor-free survival after treatments. Moreover, most patients seem to maintain a stable graft function, with no case of PSC recurrence or graft loss. Long term data on oncological outcome are scarce.

After LT, CRC represents a severe life-treating risk for patients affected by PSC and UC. Singh et al. reported an estimated CRC incidence rate of 5.8–13.5 per 1000 patient years post-transplant [36].

In a large retrospective study from the UK national registry analysing 8115 adults after LT, Rompianesi et al., confirming USA previous data [37], demonstrated that LT recipients with UC and PSC have a significantly increased risk of CRC, with a standardized incidence ratio of 7.0, when compared to LT patients with no colitis nor PSC [34]. Interestingly 23 patients out of 354 with UC and LT but without PSC had colorectal cancer with an incidence of 6.5% [34]. Also, when comparing incidence of PSC patients with or without IBD, another study demonstrated that patients with PSC-IBD had a 10-year incidence of 11.8%, whereas patients with PSC without IBD had a 10-year cumulative incidence of 2.8% [38]. In a Mayo Clinic study, when comparing these patients with the historical cohort with PSC-IBD who did not undergo LT, the rate of CRC was 4.4 higher [26]. In contrast, Goss et al. showed that CRC does not develop in this patient population more frequently than would be expected for a similar population of patients with UC [39].

**Fig. 4:**
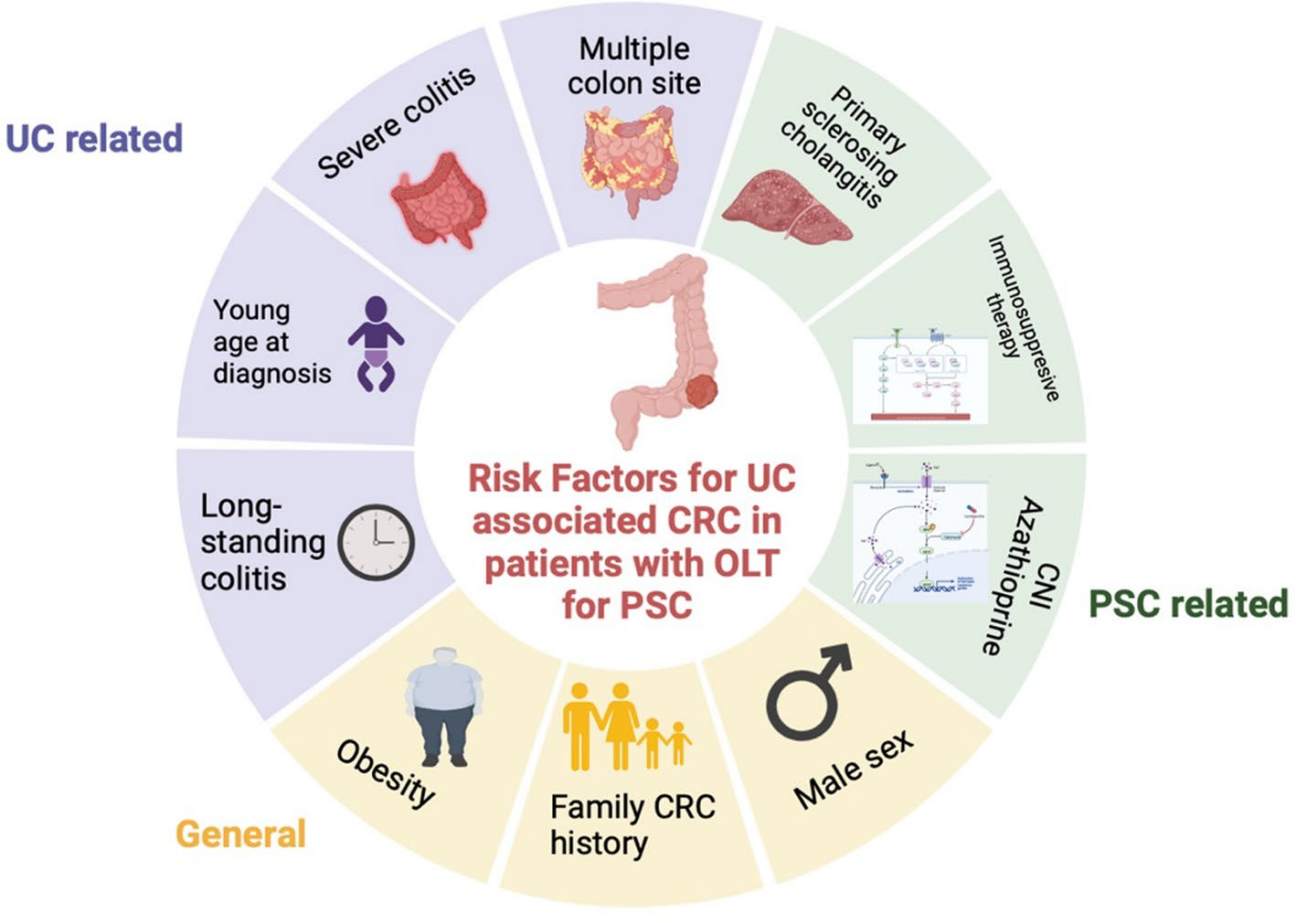
Risk factor for colorectal cancer in liver transplantation for PSC and UC

Despite the evidence that patients with PSC-UC are at high-risk of developing CRC, their clinical characteristics prevention, treatments, and oncological outcomes after tumor development are still unclear [40]. The present analysis provides an overview of the risk factors influencing de novo CRC in this population (Fig. 4).

CRC seems to occur mainly in male adults in their fifties (66%), after a long history of active colitis (over 20 years) (79%), similarly to what is seen in UC-associated CRC in the non-transplanted population [34].

The median time between LT and CRC occurrence is about 3 years, with 1/3 of the patients that developed de novo neoplasm within 2 years after the transplant. The distribution of CRC seems to be equal across all locations, but unfortunately information on tumour site was limited to a small proportion of cases (32%).

Active colitis is certainly an independent predictor of CRC, and in LT recipients is of utmost importance to evaluate the extension and severity of the inflammation using validated measurements such as the Mayo Endoscopic Score and the more recent UC Endoscopic Index of Severity [41, 42]. It is questionable if LT recipients with PSC-UC should follow the standard CRC screening recommended for the general UC population or instead they need a stricter surveillance, especially in case of active inflammation [43, 44]. Nevertheless, data from the pooled analysis, showed that the global incidence of CRC diagnosed at an early stage was 66%, suggesting that the actual surveillance programs seem to be efficacious.

The incidence of CRC is higher if compared to the general LT population, where it is up to 0.2% at 5-years and 0.8% at 10-years, with a reported mortality of 20%, representing the second most common cause of death [45]. The mid-term oncological outcomes were good, with a 3-years DFS of 75% and an overall 3-year patients’ survival of 78%, the latter being comparable to LT recipients transplanted for other indications [8]. Only one study [34] reported long-term outcomes, showing a 5-years and 10-years survival rate of 59% and 47% respectively.

At the time of tumour diagnosis, most patients were receiving triple IS therapy, based on steroids associated with any type of Calcineurin Inhibitors (CNI) -Cya or Tac- and MMF or Aza. Certainly, the post-transplant exposure to high doses of IS drugs might increase the risk of developing de novo neoplasm such as CRC. On the other hand, the same therapeutic agents are commonly used to treat UC inflammation and, therefore, it is difficult to understand their role in this scenario. In 2013, Mosli et al. showed that PSC patients treated after LT with steroids, Cya and Aza seem to provide a more benign course of UC [46]. In 2016, Safaeian et al. described an increased risk of CRC in patients treated with CyA and Aza compared with Tac and MMF, while previously Haagsma et al. found an increased risk for UC patients treated with double therapy including Tac and steroids as compared to triple therapy (Cya, Aza and steroids) [37, 47].

Regarding the use of steroids, which represents the main standing therapy in UC and PSC patients, the benefit of their prolonged use is unclear. Few studies showed that steroids reduce the inflammation, while others reported no change in the severity of the colitis, but an increased risk of steroids-related side effect [48–50]. The two most common therapeutic regimens, include a progressive withdrawal of steroids with maintenance therapy with MMF/Aza [2, 51] or a chronic administration of steroids, independently from the use of other IS drugs [49, 52]. Nevertheless, in a recent meta-analysis comparing steroids-free versus steroids-use after LT, there were no difference in terms of mortality, graft loss or infections [53].

The CNI-regimen (Cya or Tac), which nowadays represent the principal IS therapy after LT, certainly plays another crucial role in this specific population. In fact, both CNIs inhibit the calcium- and calmodulin-dependent phosphatase protein or calcineurin [54, 55] and they cause an enhanced production of TGFβ-1, which is implicated in the development of tumours [56]. On the other hand, in case of severe exacerbations of UC, the administration of intravenous Cya or oral Tac have been reported to be effective alternatives to steroids or even to total colectomy [57, 58].

As alternative, low doses of Aza demonstrated efficacy and safety in patients with chronic active UC showing an endoscopic improvement and mucosal healing, by the inhibitions of purine nucleotide synthesis and interfering with RNA synthesis/metabolism [59]. Also Aza is associated with increased risk of de novo malignancy [60].

Among IS drugs, a promising role could be represented by the Mammalian-Target of Rapamycin (m-TOR) inhibitors, which have clearly demonstrated to reduce the incidence of de novo malignancy and tumour recurrence (i.e. hepatocellular carcinoma) after LT [61]. However, from our analysis, only one patient received m-TOR inhibitors (Sirolimus, Rapamune) at the time of CRC diagnosis, and he didn’t experience tumor recurrence neither death at 12 months of follow-up. The scarce experience with m-TOR inhibitors is probably due to the recent introduction of this drugs as post-LT IS regimen and furthered experiences are needed to explore their potential role [32].

Unfortunately, none of the analysed studies provide details regarding the IS management after the diagnosis of dysplasia or CRC in PSC-UC recipients. Generally, in case of de novo neoplasm after LT, international recommendation suggests IS minimization and introduction of m-TOR inhibitors [62–69]. However, in LT patients with PSC and UC, reduction of IS might be challenging due to the underlying autoimmune disease, which could cause reactivation of UC and/or PSC and graft rejection. Chronic graft rejection might be irreversible, leading to graft loss [70]. In the current review, after tumor diagnosis all patients maintained a good graft function, except one LT recipient who died for chronic rejection after minimization of IS due to CRC [29].

In this scenario, a personalized IS management, based on patient’s clinical characteristics, should be evaluated to maintain a good graft function, and provide a low risk of de novo neoplasm. In case of CRC, CNI and steroids could be reduced or discontinued in favour of the simultaneous introduction of m-TOR inhibitors [62].

Surgical management mostly consist in proctocolectomy followed by ileo-anal pouch or terminal ileostomy [14, 71–74]. Interestingly, some authors convey that pre-LT colectomy could be beneficial in term of reducing the rates of CRC and subsequent graft failure or death [75]. In this context, colonoscopy at the diagnosis of PSC remains mandatory and should be repeated at 1–2 years interval in the patients with UC [76]. For what concern neo-adjuvant or adjuvant treatments, were scarcely reported, but it seems that there is a tendency towards a “light” approach, possibly related to the perceived fragility of LT recipients and associated IS therapy, or maybe because most tumors were detected at an early stage.

The current analysis is first hampered by the paucity of reports. Furthermore, most of the available data are not homogeneous, are often dated and sometimes incomplete.

## Conclusions

UC patients after LT for PSC shows an increased risk of CRC, especially male patients in their fifties with a long history of active colitis. CRC risk is especially high within the first few years from transplantation. Current programs of screening seem to be adequate and total proctocolectomy offer good oncological outcomes. Data on long-term cancer related survival are scarce. Tailored IS therapy might play a central role in preventing post-transplant de novo tumors and disease progression after CRC treatment and, at the same time, might increase the rate of graft survival. National and international registry are auspicial to evaluate optimal management and long-term oncological outcome.

## Data Availability

No datasets were generated or analysed during the current study.
